# Moisture Susceptibility Evaluation of Asphalt Mixtures Containing Steel Slag Powder as Filler

**DOI:** 10.3390/ma12193211

**Published:** 2019-09-30

**Authors:** Zhifeng Xiao, Meizhu Chen, Shaopeng Wu, Jun Xie, Dezhi Kong, Zhi Qiao, Changchang Niu

**Affiliations:** 1 State Key Laboratory of Silicate Materials for Architectures, Wuhan University of Technology, Wuhan 430070, China; xiaozf@whut.edu.cn (Z.X.); wusp@whut.edu.cn (S.W.); xiejun3970@whut.edu.cn (J.X.); kongde@whut.edu.cn (D.K.); 2 Inner Mongolia Comprehensive Transportation Science Research Institute Co., Ltd., Inner Mongolia 010000, China; qz20170523@yeah.net (Z.Q.); ncc924824770@163.com (C.N.)

**Keywords:** steel slag powder, asphalt, filler, adhesion, moisture susceptibility

## Abstract

The primary objective of this paper was to investigate the effect of replacing steel slag powder (SSP) with limestone filler (LF) with different contents as an inorganic anti-stripping agent on the moisture susceptibility of asphalt mixtures. Two traditional inorganic anti-stripping agents were selected for comparison, namely cement (CE) and slaked lime (SL). Apparent morphology, chemical compositions, and the particle size distribution of the four fillers were firstly studied. LF was replaced by SSP, CE, and SL with different contents, and then mixed with asphalt to prepare asphalt mortars. An 80 °C water immersion test was conducted to investigate the adhesion of asphalt mortar and aggregates, and an image analysis technique was utilized to evaluate the stripping of asphalt from the aggregates. A Marshall stability test and freeze-thaw split test were then conducted to analyze the effect of different fillers on the moisture susceptibility of asphalt mixtures. The results show that SSP contains a large amount of CaO, which indicates that SSP has a certain alkalinity. Compared with LF, SSP has a rougher surface texture and a finer particle size. Image analysis results show that the partially replacement of LF by SSP increases the asphalt coverage rate of aggregates, which means that SSP can improve the adhesion between asphalt mortar and aggregates. However, the excessive addition of SSP will result in a decrease in adhesion. The results of both the Marshall stability test and freeze-thaw split test demonstrate that CE, SL, and SSP can improve the moisture susceptibility of asphalt mixtures compared with the LF group, and that asphalt mixtures containing SSP have better moisture damage resistance than those with CE, but less such resistance than those with SL. With the increase of the amount of SSP replacing LF, the moisture susceptibility of the asphalt mixture decreases gradually. The optimum substitution amount of SSP was 25% of the total volume of fillers in this test.

## 1. Introduction

Asphalt mixtures, a superior pavement material, consist of an asphalt binder, mineral filler, and fine and coarse aggregates [1]. The mineral filler and asphalt binder are mixed to form the asphalt mortar, which bonds the aggregates into a whole to ensure the excellent pavement performance of the asphalt mixture. With the rapid development in highway construction and consequential depletion of natural resources, the search for a substitute for natural fillers has attracted increasing attention. In recent decades, more and more researchers have focused on the reuse of wastes in road pavements, such as waste tires [2], waste glass powder [3], construction and demolition wastes [4], reclaimed asphalt pavement [5], coal waste [6], and so on.

The steel industry currently produces many million tons of slags waste in the separation of molten metal, iron, and steel from oxides [7]. More than 100 million tons of steel slag are produced per year in China; however, the effective utilization rate is less than 30% [8]. The accumulation of steel slag leads to land occupation and environment pollution. Because of the characteristics of high alkalinity, rich angularity, and good wear resistance, steel slag can improve the water damage resistance and skid resistance of asphalt pavements, and has been widely used in asphalt pavement construction [9,10,11,12]. Nevertheless, most studies have focused on the application of steel slag in asphalt pavement as aggregates, and only a few researchers have investigated the feasibility of using steel slag as mineral filler in asphalt mixtures. These studies focused on the physical and chemical properties of steel slag powder (SSP) and the rheological properties of asphalt mortar, and suggested that SSP could be used as the substitute for natural mineral filler to improve the pavement performance of asphalt [13,14].

Moisture damage is one of the most common damages in the service of asphalt pavements [15]. It can be attributed to the fact that water in asphalt mixtures weakens the adhesion between the aggregate and the asphalt, as well as the cohesion in the asphalt mortar [16]. The mineralogical properties of aggregates are the key factor to maintaining the firm bond with asphalt in the presence of water. Asphalt is an acidic material which has good adhesion with alkaline aggregates such as limestone and basalt, but poor adhesion with acidic aggregates such as granite and sandstone [17]. The use of lime materials and liquid anti-stripping agents is one of the methods by which to improve the adhesion between asphalt and aggregates [18]. The liquid anti-stripping agent has good anti-stripping effect; however, its long-term performance has been questioned, because of its decomposition at high temperatures [19]. Traditional mineral anti-stripping additives, such as slaked lime (SL), cement (CE), fly ash, and flue dust have been widely shown to improve the moisture susceptibility of asphalt mixtures [20,21,22]. This can be explained by the fact that these materials contain a large number of alkaline components which react with acidic components in asphalt to form insoluble organic calcium salts, and consequently, improve the water damage resistance of asphalt mixtures [23]. SSP contains a large amount of CaO produced in steelmaking process [24], and has potential as an inorganic anti-stripping agent for use in asphalt mixtures.

In this study, the effect of using SSP as an inorganic anti-stripping agent to improve the water damage resistance of asphalt mixtures was investigated. For this purpose, SSP was added to asphalt and asphalt mixtures as an additive of asphalt or as a filler. Two traditional inorganic anti-stripping agents, namely cement and slaked lime, were selected for comparison. The surface morphology, chemical composition and particle size distribution of SSP, as well as cement, slaked lime, and limestone filler, were studied by scanning electron microscopy (SEM), X-ray fluorescence (XRF), and laser particle size analyzer (LPSA). The adhesion of asphalt and aggregate mixed with different fillers was evaluated via an 80 °C water immersion test, and an image analysis method was used to replace conventional visual analysis in order to accurately determine the asphalt stripping rate. Finally, a Marshall stability test and freeze-thaw split test were conducted to analyze the moisture susceptibility of asphalt mixtures.

## 2. Materials and Methods

### 2.1. Raw Materials

#### 2.1.1. Asphalt Binder

The conventional 80/100 penetration graded asphalt binder was used in this research; its basic properties are listed in Table 1.

#### 2.1.2. Fillers and Aggregate

**Figure 1:**
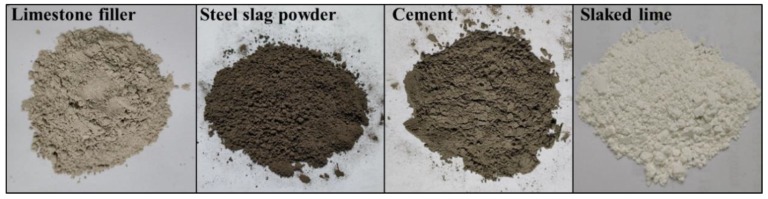
Appearances of the four fillers.

Four filler types, namely limestone filler (LF), steel slag powder (SSP), cement (CE), and slaked lime (SL) were used in this study. LF is widely used in asphalt mixtures, and is also used as a control group in this study. SSP was obtained by ball milling steel slag. CE and SL were used as traditional inorganic anti-stripping agents for comparison. The appearances and the basic properties of the four fillers are shown in Figure 1 and Table 2. As seen in Figure 1, SSP has the darkest color, which may be attributed to the presence of iron particles, as pure iron powder is black. It can be seen from Table 2 that the SSP has the highest density, i.e., 3.735 g/cm^3^, which is significantly higher than those of other fillers. This may attribute to the residual iron in SSP. The hydrophilic coefficient of the four fillers are all less than 1, which indicates that they are hydrophobic. SSP has a lower hydrophilic coefficient (0.693) compared to LF (0.716), which shows its potential for use as a filler in asphalt mixtures.

In this investigation, granite was selected as coarse and fine aggregates; granite is a typical acidic aggregate with poor adhesion to asphalt and is vulnerable to water erosion. The aggregate grading was selected based on the Test Methods of Aggregate for Highway Engineering (JTG E42-2005), with a maximum nominal size of 19 mm.

#### 2.1.3. Preparation of Asphalt Mortar

Relevant research has shown that the dosage of cement and slaked lime should be 20%–40% of the filler mass when they were used as anti-stripping agents to replace the mineral filler. However, the density of the four fillers differs greatly, so it is more appropriate to use a volume replacement ratio. In this paper, LF was replaced by SSP, CE, and SL in different dosages (by volume). Twenty-five percent of the substitution of LF by volume was used. The group numbers and filler constitution are shown in Table 3. The four fillers were dried at 105 °C for 3 h and then added to the asphalt, according to a powder-to-binder ratio of 1:1. The asphalt and fillers were mixed for 10 min using a high-speed shearing machine (3000 r/min, 135 °C). Due to the large difference in density between the fillers and the asphalt, the asphalt mortars will be segregated during storage, and the fillers will gradually sink to the bottom, resulting in inhomogeneity. Therefore, it is necessary to mix the asphalt mortars again before using them.

### 2.2. Experimental Methods

**Figure 2:**
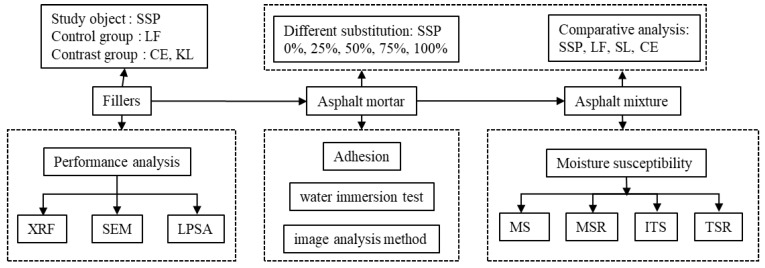
Framework of experiment procedure.

The framework of the experimental procedure is illustrated in Figure 2. The basic properties of the four fillers were firstly studied. Image analysis was performed after a water immersion test to quantify the adhesion quality between the asphalt mortar and aggregate; additionally, a Marshall stability test and freeze-thaw split test were undertaken for moisture susceptibility assessment. Several substitution ratios of LF by SSP were compared in order to find a good candidate regarding the initial objective.

#### 2.2.1. Fillers and Aggregate Characteristics

The properties of fillers will affect the performance of the asphalt mortar, which in turn affects the moisture damage resistance of the asphalt mixture. Therefore, some properties of the four fillers were analyzed. An X-ray fluorescence spectroscopy (XRF, PANalytical B.V., Amsterdam, The Netherlands) was used to determine the elemental composition of the four fillers. Scanning electron microscopy (SEM, NEC Electronics Corporation, Tokyo, Japan) was used to characterize the surface characteristics of the four fillers. The particle size is an important parameter for evaluating fillers, and its distribution has a significant impact on the performances of asphalt mortar and its mixtures. A Malvern Mastersizer 2000 laser particle size analyzer (LPSA, Malvern Instruments, Malvern, UK) was used to determine the particle size distribution.

#### 2.2.2. Marshall Mixtures Design Method

**Figure 3:**
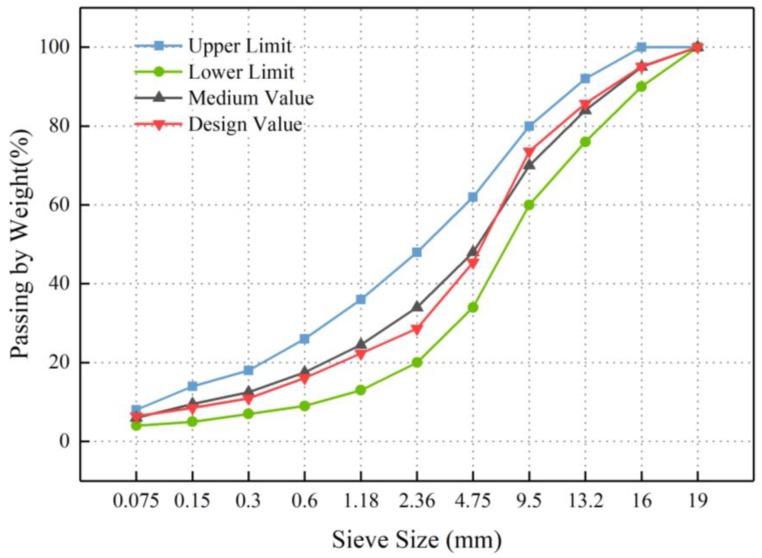
Gradation curve of AC-16.

The Marshall mixtures design method, as specified in Technical Specification for Construction of Highway Asphalt Pavements (JTG F40-2004 [26]), was used in this investigation. Because the aggregates have a maximum nominal size of 19 mm, AC-16 aggregate gradation was chosen to prepare the hot mix asphalt (HMA). The gradation curve of AC-16 is shown in Figure 3, in which the upper and lower limits of the gradation curves followed the requirements of JTG F40-2004. The optimum content of the asphalt binder was determined by the volume parameters (e.g., air void, voids in mineral aggregate, and voids filled with asphalt). The content of mineral fillers was 5% by weight of aggregate. According to the designed filler type in Table 3, SSP, CE, and SL were used to replace LF to prepare Marshall specimens. In order to ensure the same volume performance of the Marshall specimens, the substitution amount in this paper was measured by the volume ratio considering the difference in the specific gravities of the four fillers.

All the asphalt mixtures with different fillers were prepared at a temperature of 160 °C and compacted at a temperature of 150 °C. Samples were compacted using a Marshall hammer with 75 blows on each side. The samples for the freeze-thaw split test were only compacted 50 times to ensure a higher void ratio, according to the requirements of the specification of JTG E20-2011 [25].

#### 2.2.3. Adhesion of Asphalt Mortar and Aggregate

The water immersion test is, comparatively, a quick and simple method to investigate the adhesion of asphalt and aggregate in water. This test was carried out on loose mixtures in accordance with JTG E20-2011. First, 100 g of aggregates were mixed with 5.5g ± 0.2 g of asphalt mortar at 160 °C. Then, the mixtures were moved onto a glass plate and cooled at room temperature for one hour. Next, the loose mixtures were put into water at 80 °C for one hour. The stripping percentage of the samples which had been cooled to room temperature was evaluated through visual observation. However, the immersed method is greatly influenced by the judgment of the operator. In order to improve the accuracy of the judgment, an image analysis method was employed to convert the test from a qualitative rating to quantitative evaluation [27].

The aforementioned immersed mixtures were cooled to room temperature and then transferred to a water-filled, white porcelain bowl. Digital images were taken of these mixtures using an external light source. The above steps were just to get a clear image of the mixtures. The next step was to process the image using the Image J software. The color images were imported into the Image J software (Version 1.52, National Institutes of Health, MD, USA) and converted into 8-bit gray scale image; then, the image’s threshold was adjusted to match the surface area of the mixtures and the asphalt coating area. The surface area of the mixtures and the asphalt coating area can be calculated through the statistical function of the software, and the area here is expressed by the software as the total number of black pixels. The asphalt coating rate (ACR) can be calculated by dividing the asphalt coating area by the surface area of mixtures as shown in Equation (1).

(1) $$ACR=\frac{A_{a}}{A_{m}}\times 100\%$$

where *ACR* = The asphalt coating rate, *A_a_* = the asphalt coating area, and *A_m_* = the surface area of mixtures.

#### 2.2.4. Moisture Damage Resistance of Asphalt Mixtures

Eight Specimens were prepared using the method introduced in 2.2.2 and divided into two groups, in accordance with JTG E20-2011.Two kinds of water stability tests were conducted. The first was the Marshall stability test. The two groups of samples were immersed in 60 °C water for 48 h and 0.5 h respectively. The Marshall stability ratio (MSR) of the two groups of the samples was measured. The specific calculation formula is shown in Equation (2).

(2) $$MSR=\frac{MS_{m}}{MS_{d}}\times 100$$

where *MSR* = Marshall stability ratio (%), *MS_m_* = average Marshall stability of samples after 48 h immersion (MPa), and *MS_d_* = average Marshall stability of samples under dry conditions (MPa).

The second one is the freeze-thaw split test. A group of samples was saturated in a vacuum container with a vacuity of 97.3–98.7 kPa for 15 min. Then, the saturated specimens were placed in a freezer at −18 °C for 16 h and then immersed in a water bath at 60 °C for 24 h. After that, both the unconditioned and conditioned specimens were soaked in a 25 °C water bath for 2 h. The indirect tensile strength (ITS) of the samples was tested, as shown in Equation (3). The moisture resistance of the samples is represented using the tensile strength ratio (TSR), which can be determined by Equation (4).

(3) $$R_{T}=\frac{0.006287P_{T}}{h}$$

where *R_T_* = tensile strength (MPa), *P_T_* = peak load (N), and *H* = specimen height (mm).

(4) $$TSR=\frac{\bar{R_{TM}}}{\bar{R_{TD}}}\times 100$$

where *TSR* = tensile strength ratio (%), $\bar{R_{TM}}$ = average tensile strength of the moisture conditioned subset (MPa), and $\bar{R_{TD}}$ = average tensile strength of the dry subset (MPa).

## 3. Results and Discussion

### 3.1. Filler Characteristics

#### 3.1.1. Surface Characteristics

**Figure 4:**
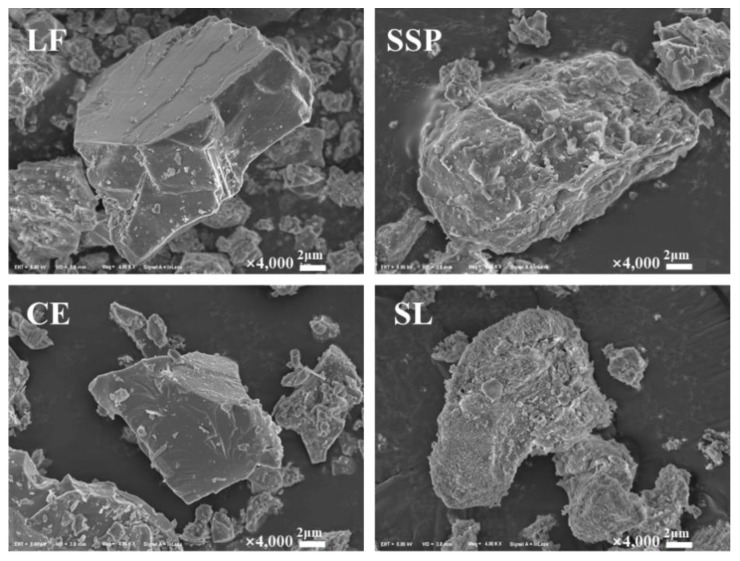
SEM images of the four fillers.

SEM images of the four fillers are shown in Figure 4. It can be seen that these four fillers exhibit different particle sizes, particle shapes and surface textures. The SSPs have a rougher surface texture with obvious lumps or holes, which is similar to the SL group. The fillers with complex surface textures usually have higher surface activity for their higher capacity of adsorbing the asphalt by the filler particles [28]. The complex surface textures could lead to an increase in asphalt coating the aggregate, which may affect the stripping resistance of mixtures [29]. Compared to SSP and SL, the surface of LF and CE is smoother, without obvious attachments, and their shapes are regular and have an obvious angularity. Therefore, SSP and SL have a larger contact area with the asphalt, which consequently improves the stiffness of the asphalt mortar and the adhesion between the asphalt mortar and the aggregate.

#### 3.1.2. Chemical Compositions

The XRF results of the four fillers are shown in Table 4. It can be seen that all fillers contain a large amount of CaO components. However, SSP contains a certain amount of Fe_2_O_3_, which is the residual iron that has not been removed during the steel-making process. Consequently, the density of SSPs density is higher than that of other fillers. CaO and Ca(OH)_2_, as recognized active ingredients, play an important role in improving the adhesion between the acid aggregate and the asphalt [30]. Comparing the CaO content of the four fillers, it can be found that that of SSP is 36.075%, which is lower than that of the other three fillers. SL contains the largest content of CaO, i.e., 73.256%. LF contains 55.539% CaO; however, the main mineral phase of LF is CaCO_3_. A large amount of CO_2_ is released during the XRF test, resulting in a high loss on ignition. The ratio of CaO content to loss on ignition in the XRF data of LF is about 1.33, which is similar to the relative molecular mass ratio (1.27) of CaO and CO_2_ in CaCO_3_, indicating that the main component of LF is CaCO_3_. The literature shows that both quicklime (the main chemical component is CaO) and hydrated lime (the main chemical component is Ca(OH)_2_) can improve the adhesion of acidic aggregates with asphalt, while CaCO_3_ is ineffective [31]. The effective CaO content of the four fillers ranges from large to small, namely, SL, CE, SSP, and LF. The content of SiO_2_ is 67% for the granite aggregate, indicating that granite is an acid aggregate.

#### 3.1.3. Particle Size Distributions

**Figure 5:**
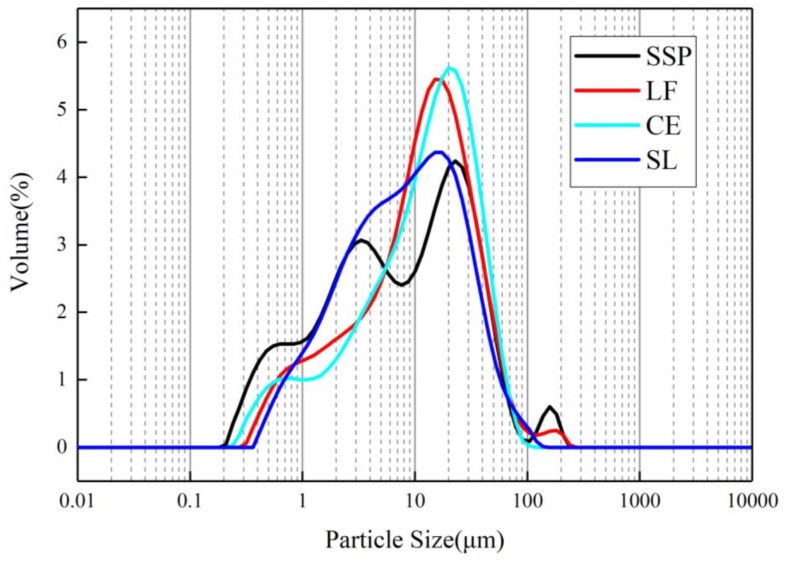
Particle size distributions of the four fillers.

The particle size distributions of the four fillers are shown in Figure 5. The particle size distribution of SSP is relatively dispersed, and there are more powders in a certain size range, while those of the other fillers are relatively uniform. This can be explained by the fact that SSP contains more impurities and a part of the iron substance, and the iron has the characteristics of high density and good wear resistance, which result in large particle size dispersion. CE and LF have similar particle size distributions, and 80% of the particles are between 1.4 and 41 μm. The particle size of SL is obviously fine, and SL contains more particles in the range of 1–10 μm compared with CE and LF. The relevant parameters of particle size are listed in Table 5. It can be seen from Table 5 that the specific surface area of the SSP is significantly larger than that of LF, CE, and SL, i.e., 2.41 m^2^/g. The larger specific surface area increases the contacting surface between SSP and asphalt, which affects the performance of the asphalt mortar, and eventually affects the performance of the mixtures.

### 3.2. Adhesion of Asphalt and Aggregate

**Figure 6:**
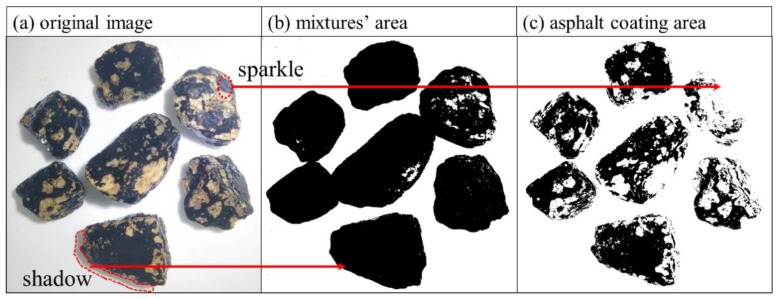
Image analysis method.

**Figure 7:**
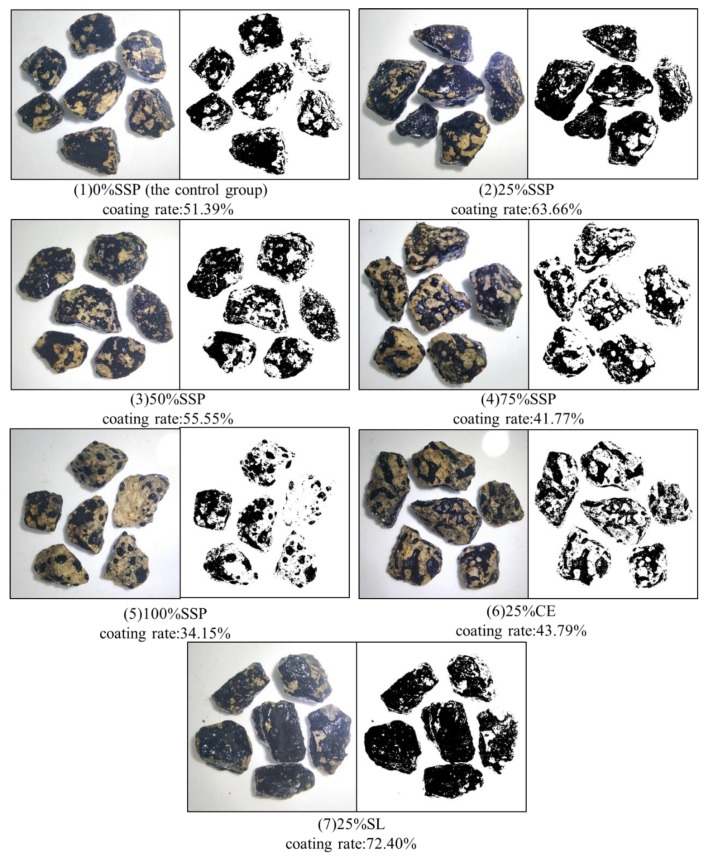
Image processing results and asphalt coverage rate.

The sample with 0% SSP was taken as an example, and Figure 6a shows a color image of the mixtures after water immersion treatment. Some representative samples were only selected for observation. Figure 6b is an image of the mixtures after the threshold adjustment, in which the black part indicates the area of the mixtures. Figure 6c shows the coating area of asphalt; in this test, any thin, brownish, translucent areas are considered to be fully coated [27]. The results of the asphalt coating rate are shown in Figure 7 for the asphalt mixtures with various fillers after water immersion treatment.

It should be noted that the results of asphalt coating rate are lower than the actual values. The digital color photographs were taken of the mixture under an external light source. Light makes the mixture more clearly visible from the background, but it also produces shadows and sparkle. On the one hand, the darkened area will inevitably be included in the total area of the mixtures (Figure 6a), although some unrecognized areas with similar gray values to the background offset the increase in total area (Figure 6b). On the other hand, the sparkle of asphalt surface will be recognized by the software as white pixels, as shown in Figure 6c. These two factors lead to lower coverage results, but this error will not have a significant impact on the comparison of the stripping resistance of the various mixtures. Compared with traditional visual observation, image analysis is a more objective method.

As shown in Figure 7, the adhesion between asphalt mortar and aggregate is greatly affected by the different fillers. The whole LF group (namely the control group) retains a 51.39% asphalt coating rate after water immersion for 1 h at 80 °C. The 25% SL group exhibits a best anti-stripping performance, with a 72.4% asphalt coverage rate, compared to an asphalt coating rate of 43.79% for the 25% CE group. SSP can also significantly improve the stripping resistance of the mixtures. The asphalt coverage rates of the mixtures at 25% and 50% substitution are 63.66% and 55.55%, respectively. However, the excessive amount of SSP is detrimental to the adhesion of asphalt to the aggregate, and the asphalt coating rates under 75% and 100% substitution are 41.77% and 34.15%, respectively. It is speculated that the iron and its oxides in steel slag powder may have an adverse effect on adhesion. The hydration reaction of some active ingredients in SSP with water during the water immersion process weakens the adhesion between asphalt and the aggregate, which can also explain the low coating rate of the 25% CE group.

### 3.3. Moisture Damage Resistance of Asphalt Mixtures

#### 3.3.1. Marshall Stability and Marshall Stability Ratio

Four replicates were tested in accordance with JTG E20-2011 for each type of mixture with various fillers. The optimum asphalt content of the 100% SSP group was 4.9%, and that of other groups was 4.8%. The Marshall stability ratio (MSR) and tensile strength ratio (TSR) are presented in Table 6. The volumetric properties, such as average volume of air void (VV) and voids in mineral aggregate (VMA), are also presented in Table 6.

**Figure 8:**
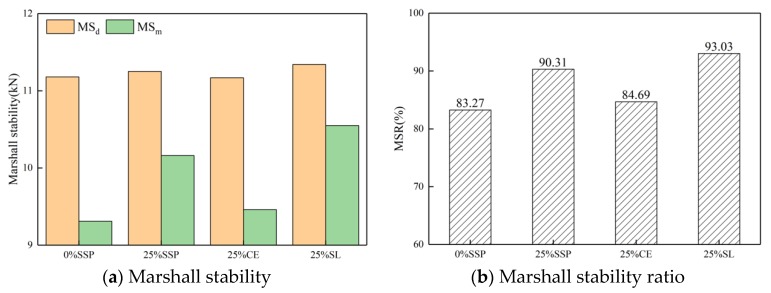
MS and MSR of samples with different fillers with the same substitution.

**Figure 9:**
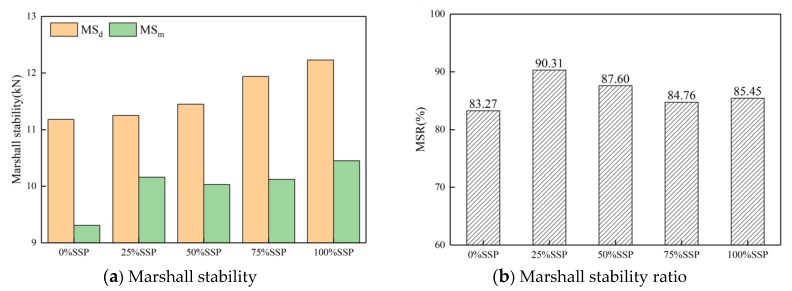
MS and MSR of samples with different substitution of SSP.

The results of the Marshall stability test are shown in Figure 8 and Figure 9. Figure 7 presents a comparison of MS and MSR of samples with different fillers with the same substitution. When SSP, CE, and SL replaced LF with the same 25% substitution amount, the Marshall stability of the samples was basically unchanged (Figure 8a), indicating that the type of filler has little effect on the Marshall stability of samples in dry conditions. After water immersion treatment, the SL significantly improved the Marshall stability of the immersed samples. The immersion stability increased by 13.3% compared with whole LF group, while SSP and CE only increased by 9.1% and 1.6%, respectively. Comparing the residual stability (Figure 8b), it was found that the highest residual stability was 93.03% for the mixtures mixed with SL, and then for the SSP group, whose residual stability was 90.31%. Cement, as a commonly-used anti-stripping agent in asphalt pavements, had little effect on improving water stability in this experiment.

Figure 9 shows the MS and MSR of samples with different substitution of SSP. In dry conditions, with the increase of substitution amount of SSP, the Marshall stability was greatly improved. The Marshall stability value of the mixtures with 100% SSP was 9.39% higher than the 0% SSP group (Figure 9a). However, this growth trend did not occur in the moisture condition subset. The value of MS did not increase regularly as it did in the drying group, but remained basically unchanged. As a result, the residual stability of the mixtures decreased gradually with the increase of the substitution amount of SSP (Figure 9b). It is speculated that the volume expansion caused by the reaction of f-CaO in the SSP with water destroyed the stability of the mixtures, and offset the reinforcing effect of the SSP on the water stability of the mixtures [31].

#### 3.3.2. Indirect Tensile Strength and Tensile Strength Ratio

**Figure 10:**
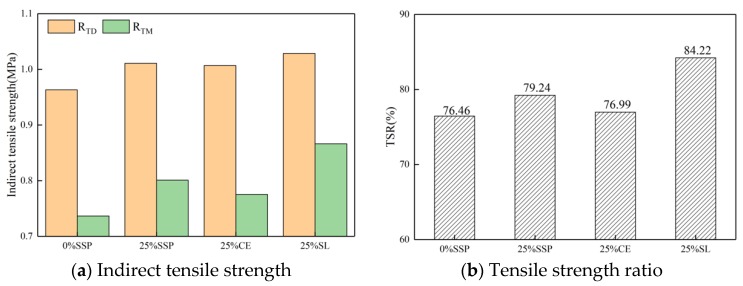
ITS and TSR of samples with different fillers with the same substitution.

**Figure 11:**
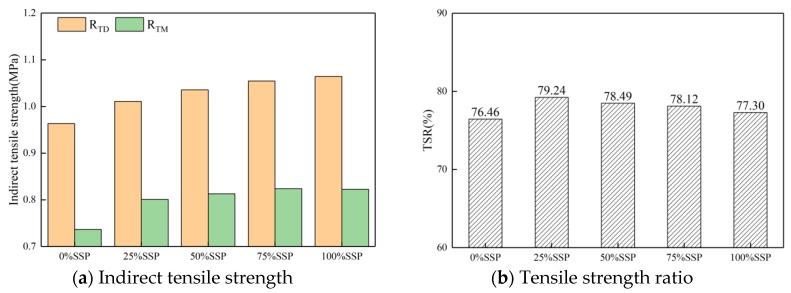
ITS and TSR of samples with different substitution of SSP.

Figure 10 and Figure 11 show the results of freeze-thaw split test; the results were similar to those of the Marshall stability test. The addition of SSP, CE, and SL can improve the values of ITS and TSR of asphalt mixtures compared to the whole LF group, in both dry and wet conditions. The 25% SSP group had a moderate value of TSR and ITS, which was lower than the 25% SL group but higher than the 25% CE group. The increase of substitution amount of SSP under drying condition had a positive effect on ITS value of mixtures, but little effect on the ITS value of the mixtures under moisture conditions, which led to the decrease of TSR. Even if the value of MSR and TSR gradually decreased with the increase of the SSP substitution amount, the value of MSR and TSR of the mixture under the 100% SSP substitution amount are still higher than those of the control group, indicating that the incorporation of SSP is beneficial to the water stability of asphalt mixtures, regardless of the amount of substitution. Both the results of the Marshall stability test and the freeze-thaw split test show that there is an optimum substitution amount of SSP, i.e., 25%.

By analyzing the correlation between the adhesion of asphalt mortar with aggregate and moisture damage resistance, it can be found that the results of a water immersion test for asphalt mixtures with different fillers basically reflect the results of moisture damage resistance. Loose mixtures with low stripping rates usually have better moisture damage resistance. The difference is that the excessive steel slag powder (such as 75% and 100% substitution) will make the adhesion lower than the control group, and that the steel slag powder is beneficial the water stability of the mixture, regardless of the amount of substitution.

## 4. Conclusions

In this study, the basic properties of steel slag powder (SSP) and other three inorganic fillers, namely limestone filler (LF), cement (CE), and slaked lime (SL), were analyzed. Meanwhile, the effect of the four fillers on asphalt-aggregate adhesion and the moisture damage resistance of asphalt mixtures were studied. Based on the results above, the following conclusions can be drawn.

(1) The effective CaO content of SSP is higher than that of limestone filler but lower than that of cement and hydrated lime, indicating that it is a high alkalinity slag filler with potential activity. However, the large amount of iron contained in SSP may have adverse effects on the moisture damage resistance of asphalt mixtures. The SSP has a rougher surface texture with obvious lumps or holes, and also has a finer particle size compared with LF and CE. The specific surface area of SSP is significantly higher than that of the other three fillers, which means that its contact area with asphalt is larger, and consequently, that it affects the performance of the asphalt mixtures.
(2) SSP and SL can improve the adhesion between asphalt and aggregate under the same substitution, but CE has the opposite effect. The 25% SL group exhibits the best anti-stripping performance with 72.4% asphalt coverage rate, compared to an asphalt coating rate of 43.79% for the 25% CE group; this value is 51.39% for the control group (0% SSP group). Under the same substitution, the asphalt mixtures with SSP have a 63.66% asphalt coverage rate after water immersion treatment, which will gradually decrease with the increase of the substitution amount. The asphalt coverage rate will be reduced to 34.15% when SSP replaces LF by 100%.
(3) It was found that the replacement of LF by CE, SL, and SSP with the same substitution amount (25%) had little effect on Marshall stability and indirect tensile strength without water treatment, and that the increase of SSP replacement amount promotes the growth of MS and ITS. After the water treatment, the effect of CE, SL, and SSP on improving water damage resistance will become more prominent, and the three fillers can resist the reduction of water on the performance of mixtures, which is attributed to their improvement of asphalt-aggregate adhesion. The effect of slaked lime on improving water damage resistance is the most obvious. Compared with the control group (0% SSP group), the value of MSR and TSR of the mixtures mixed with SL were improved by 11.72% and 10.15%, respectively. The effect of cement on improving water damage resistance was slightly worse. SSP had a positive effect on improving the water stability of asphalt mixture, but with the increase of substitution amount, MSR and TSR decreased; the optimal dosage is 25%. Nevertheless, the addition of SSP improved the water damage resistance of asphalt mixtures compared to the whole LP group, regardless of the amount of substitution.
(4) Both the water immersion test and the water stability test indicated that the SSP could replace part of the limestone filler as a filler, but that the substitution amount should not be too great. In this experiment, the best substitution amount of SSP was found to be 25% of the total volume of fillers.

## Figures and Tables

**Table 1:** Basic properties of asphalt.

Properties	AH-90 Asphalt	Standard [25]
Density (g/cm^3^)	1.032	T 0603-2011
Penetration (25 °C, 0.1 mm)	85	T 0604-2011
Softening point (°C)	46	T 0606-2011
Ductility (10 °C, cm)	165	T 0605-2011
Viscosity (135 °C, Pa·s)	0.35	T 0625-2011
flash point (°C)	278	T 0611-2011

**Table 2:** Basic properties of four powders.

Properties	LF	SSP	CE	SL
Density (g/cm^3^)	2.691	3.735	3.125	2.24
Hydrophilic coefficient	0.716	0.693	0.746	0.578
Water content (%)	0.423	0.781	0.525	0.137

**Table 3:** Sample numbers and filler constitution of asphalt mortars.

Sample Numbers	0% SSP	25% SSP	50% SSP	75% SSP	100% SSP	25% CE	25% SL
Filler constitution	100% LF	25% SSP + 75% LF	50% SSP + 50% LF	75% SSP + 25% LF	100% SSP	25%CE + 75% LF	25%SL + 75% LF

**Table 4:** Chemical compositions of the four fillers and granite aggregate.

Components	CaO	Fe_2_O_3_	SiO_2_	MgO	MnO	Al_2_O_3_	SO_3_	LOI	Others
Steel slag powder	36.075	26.276	17.163	6.567	4.876	3.650	0.639	0.365	4.389
Limestone filler	55.539	0.094	1.769	0.534		0.166	0.036	41.838	0.024
Cement	58.111	3.212	20.663	2.624	0.148	6.135	3.253	4.045	1.809
Slaked lime	73.256		0.693	0.752		0.120		24.927	0.252
Granite	2.678	3.573	67.065	1.064	0.091	14.976	0.033	1.287	9.233

**Table 5:** Particle size parameters of the four fillers.

Particle Size Parameters	SSP	LF	CE	SL
d (0.1)	0.814	1.464	1.468	1.549
d (0.5)	8.262	12.785	14.046	8.981
d (0.9)	41.283	41.163	41.205	35.561
Average particle size of surface area (μm)	2.485	4.195	3.956	4.034
Average particle size of volume (μm)	17.899	19.000	18.287	14.998
Specific surface area (m^2^/g)	2.41	1.43	1.52	1.49

**Table 6:** Water resistance results and volumetric properties of different type of mixtures.

Mix Type	Number of Compaction	VV (%)	VMA (%)	MSR	TSR
0%SSP	75	5.10	15.80	83.27	76.46
	50	6.55	17.05		
25%SSP	75	5.17	15.83	90.31	79.24
	50	6.60	17.13		
50%SSP	75	5.15	15.83	87.60	78.50
	50	6.63	17.13		
75%SSP	75	5.11	15.81	84.76	78.12
	50	6.34	16.90		
100%SSP	75	5.13	15.83	85.45	77.30
	50	6.57	17.10		
25%CE	75	5.12	15.82	84.69	77.00
	50	6.45	17.00		
25%SL	75	5.18	15.86	93.03	84.22
	50	6.63	17.17		
